# Origin of TAA Genes in Charophytes: New Insights into the Controversy over the Origin of Auxin Biosynthesis

**DOI:** 10.3389/fpls.2017.01616

**Published:** 2017-09-20

**Authors:** Facundo Romani

**Affiliations:** Instituto de Agrobiotecnología del Litoral, Universidad Nacional del Litoral, CONICET, Centro Científico Tecnológico Santa Fe Santa Fe, Argentina

**Keywords:** TAA1, auxin biosynthesis, hormone evolution, charophytes, klebsormidium

There is a current controversy around the origin of auxin biosynthesis genes in plants. Auxin is one of the most important plant hormone and its major biosynthesis pathway implies the tryptophan aminotransferase (TAA) and flavin containing monooxygenases (YUCCA) gene families (Mashiguchi et al., 2011). The hormone is present in all land plants and also in charophycean algae. Recently, it was demonstrated that algae also respond to auxin (Stirk et al., 2013; Ohtaka et al., 2017), but the presence of the biosynthesis genes in this lineage is still not clear.

A few years ago, Yue et al. (2014) claimed they failed to found TAA and YUCCA genes in charophytes, the most closer lineage to land plants (Delwiche and Cooper, 2015). Later, Wang et al. (2014) found TAA and YUCCA homologs in *Klebsormidium nitens* (formerly named *K. flaccidum*) and proposed that the biosynthesis pathway exists in green algae. Few months later, Turnaev et al. (2015) published a letter in response to the Wang's work, pointing out some failures in the analysis and proposing that TAA genes are not present in algae.

In this context, it is important to note presence of YUCCA genes in charophytes is not questioned. The controversy is the TAA-like gene identified in *K. nitens* (kfl0051_0080) is the only reported case in charophytes and no homologs have been found in many others sequenced transcriptomes from charophytes. This suggests two possibilities, either this gene is not conserved or it came from a contamination. Moreover, the protein sequence of kfl0051_0080 also contains other three domains upstream the putative TAA domain and is much larger than the TAA genes in land plants. Turnaev et al. propose the gene actually belongs to the TAA-related family of alliinases and it was originated from a different horizontal gene transfer (HGT) event (Turnaev et al., 2015).

The issue about which clade kfl0051_0080 belongs has been responded by Wang et al. in a posterior letter (Wang et al., 2016), making clear that the gene is basal to both clades. According to Wang, alliinases and TAA genes would had a common evolutionary origin and arose from an ancient gene duplication event occurred after the divergence of *K. nitens* and before plants adaptation to land.

Recently, new transcriptomes from charophytes were released, providing new tools to respond to this controversy. Given this background and using sequences from the 1 KP project (Matasci et al., 2014) and The Green Algae Tree of Life project (Cooper and Delwiche, 2016) we can reanalyze the question about the origin of TAA genes in streptophytes.

In the new datasets, there are at least five new species in charophycean algae mainly from the Zygnematophyceae class where a TAA-like gene is present. In the phylogenetic analysis, these sequences belong to a monophyletic clade that include kfl0051_0080 and before the diversification of alliinases and TAA genes (Figure 1). This solves the first controversial issue about the absence of homologs in other charophytes species, but there are still other issues to address.

**Figure 1:**
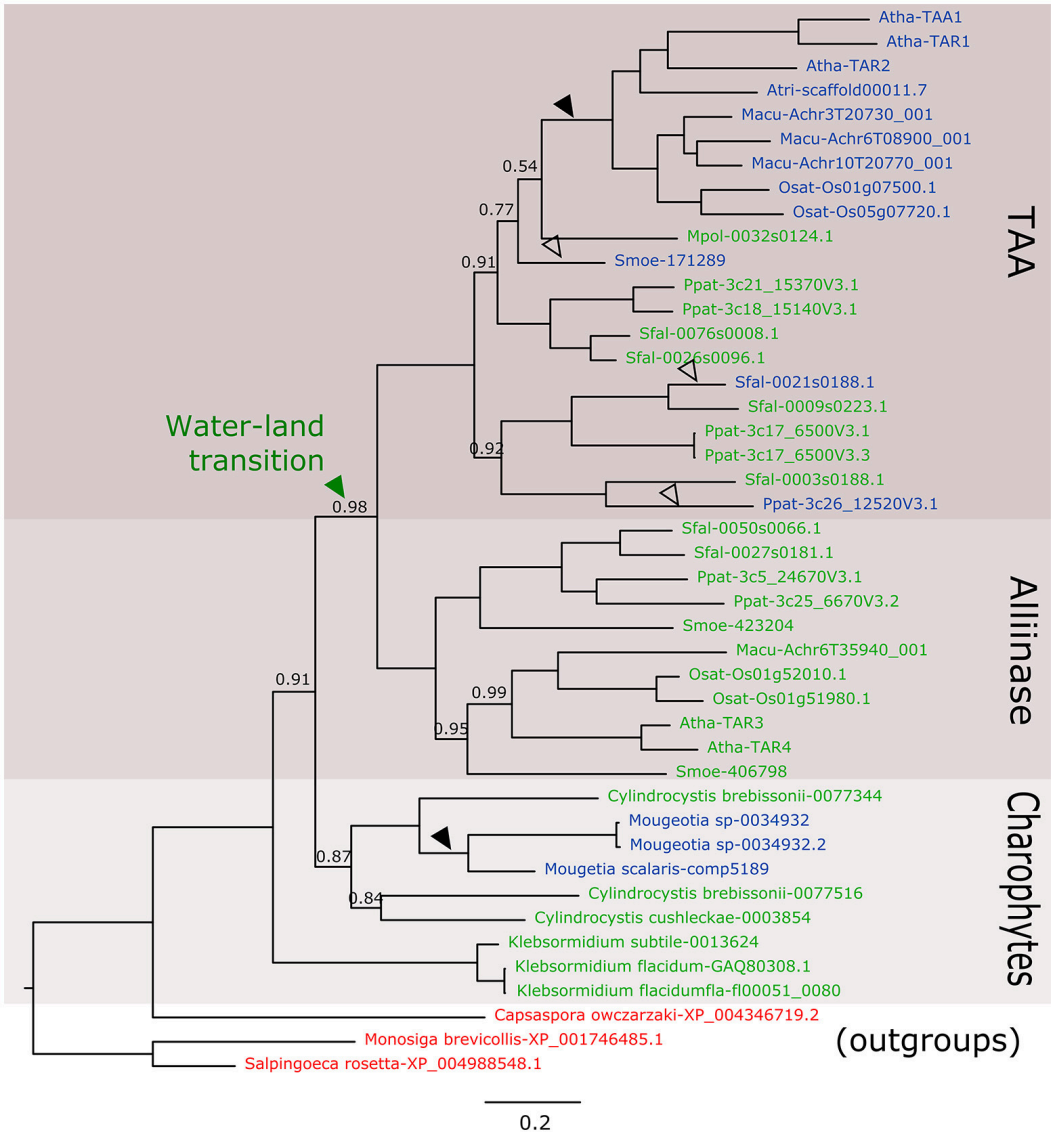
Phylogenetic relationship of TAA-like genes from Streptophytes. Nucleotide sequences of green algae species were retrieved from 1 kp (Matasci et al., 2014) and Tree of Life Project (Cooper and Delwiche, 2016) charophytes transcriptomic assemblies and using AtTAA1 protein sequence as query for tBLASTn search. In total, 36 transcriptomic experiments were used and sequences with low similarity or with more than 50% gaps in the alignments were eliminated. Putative protein sequences and reference sequences were aligned using the MUSCLE method implemented in MEGA6 (Tamura et al., 2013) and non-significant positions were manually eliminated. The phylogenetic tree was constructed using MrBayes (Ronquist et al., 2012) with *invgamma* rate variation. 11.4 million generations were run until 1E-3 mean standard deviation was obtained. Number in nodes indicates Bayesian posterior probability values, nodes have a probability value equal to 1 are not shown. The scale bar indicates the genetic distance. Reference sequences were obtained from Phytozome (Goodstein et al., 2012). Non-plant sequences in red were retrieved from NCBI and used as outgroups to root the tree similarly to Wang et al. (2014). Sequences in green contain an alliinase domain, sequences in blue do not have the alliinase domain. Black arrows indicate the position of reliable loss events of the alliinase domain and white arrow indicate the less reliable ones. Atha, *Arabidopsis thaliana*; Osat, *Oryza sativa*; Macu, *Musa acuminate*; Atri, *Amborella trichopoda*; Ppat, *Physcomitrella patens*; Some, *Sellaginella moeleindorfii*; Sfal, *Sphagnum fallax*.

In the first place, the presence of another two domains in kfl0051_0080 homologous to Arabidopsis SYN1 seems to be an error in the transcript assembly V1.0 of *K. nitens* and it is not included in V1.1 (NCBI) or any other assemblies (Cooper and Delwiche, 2016). Furthermore, the presence of an alliinase domain in TAA-like genes is not a fact that only occurs in the alliinase clade and it happens also in the TAA clade of the bryophytes *Sphagnum fallax, Physcomitrella patens*, and *Marchantia polymorpha*. In our results, it is clear that there are sequences evidently belonging to the TAA clade that also contains the alliinase domain, making the idea of different HGT events less parsimonious. The elimination of this domain occurred at least twice during evolution (Figure 1). However, we still do not know much about the function of TAA genes in basal land plants. In these plants, it has been proposedthat most of the auxin is produced by a Trp-independent pathway not involving a TAA gene (Sztein et al., 2000). In *M. polymorpha*, Eklund et al. (2015) showed a functional Trp dependent auxin biosynthesis pathway (involving TAA and YUCCA) that contributes to the auxin pool and is necessary for proper development of the gametophyte. In this case at least, the alliinase domain present in the unique TAA gene in Marchantia does not seem to affect the biosynthetic activity. More experiments, as *in vitro* biochemical characterization or transcromplementation, could give more certainties about the role of the gene in Marchantia and in other non-seed plants development.

The publication of new transcriptomes in green algae opens the door to many fundamental findings in plant evolution during the transition to land. These new data is enough to respond to two of the main criticism to the origin of TAA in charophycean algae, the poor conservation into different species and the presence of other domains in kfl0051_0080. The explanation for a common origin of alliinases and TAA genes is much more parsimonious than the alternatives and data presented here support this idea. The fact that TAA genes are only expressed in specific conditions could explain why they were not detected in transcriptomes from charophytes taxa as Charophyceae (e.g., Nitella), positioned between Klebsormidiales and Zygnematales (Ke et al., 2015). Even in the case that the gene in Klebsormidiales was lost during evolution, a HGT event in Zygnematales is a possible alternative. These species are also evolutionary much closer to land plants (Wodniok et al., 2011; Timme et al., 2012) than *K. nitens* and provide an alternative evidence to affirm that the enzyme is present in green algae and conserved during the transition to land, together with YUCCA genes (Wang et al., 2014). In conclusion, these results confirm that the origin of the main auxin biosynthesis pathway originated in charophycean algae, more than 500 million years ago.

There are many things that we still do not know about auxin in green algae. The conservation of the signaling pathway including the TIR1/AFB receptor and the AUX/IAA repressor protein is not completely clear. In a recent work Ohtaka et al. (2017), showed a primitive auxin response in *K. nitens* lacking both proteins. The presence of a true homolog to the TAA gene in green algae is not trivial and was the cause of a big controversy. This also suggests possible primitive signaling pathway. The evolution of the auxin system in modern plants is much more complex than we thought and requires many others components working together. Further research is necessary to elucidate how these components were incorporated into plant genomes.

## Author contributions

The author confirms being the sole contributor of this work and approved it for publication.

### Conflict of interest statement

The author declares that the research was conducted in the absence of any commercial or financial relationships that could be construed as a potential conflict of interest.
